# White Matter Integrity, Creativity, and Psychopathology: Disentangling Constructs with Diffusion Tensor Imaging

**DOI:** 10.1371/journal.pone.0009818

**Published:** 2010-03-22

**Authors:** Rex E. Jung, Rachael Grazioplene, Arvind Caprihan, Robert S. Chavez, Richard J. Haier

**Affiliations:** 1 Mind Research Network, University of New Mexico, Albuquerque, New Mexico, United States of America; 2 Department of Neurosurgery, University of New Mexico, Albuquerque, New Mexico, United States of America; 3 Department of Psychology, University of New Mexico, Albuquerque, New Mexico, United States of America; University of Granada, Spain

## Abstract

That creativity and psychopathology are somehow linked remains a popular but controversial idea in neuroscience research. Brain regions implicated in both psychosis-proneness and creative cognition include frontal projection zones and association fibers. In normal subjects, we have previously demonstrated that a composite measure of divergent thinking (DT) ability exhibited significant *inverse* relationships in frontal lobe areas with both cortical thickness and metabolite concentration of N-acetyl-aspartate (NAA). These findings support the idea that creativity may reside upon a continuum with psychopathology. Here we examine whether white matter integrity, assessed by Fractional Anisotropy (FA), is related to two measures of creativity (Divergent Thinking and Openness to Experience). Based on previous findings, we hypothesize inverse correlations within fronto-striatal circuits. Seventy-two healthy, young adult (18–29 years) subjects were scanned on a 3 Tesla scanner with Diffusion Tensor Imaging. DT measures were scored by four raters (α = .81) using the Consensual Assessment Technique, from which a composite creativity index (CCI) was derived. We found that the CCI was significantly inversely related to FA within the left inferior frontal white matter (t = 5.36, p = .01), and Openness was inversely related to FA within the right inferior frontal white matter (t = 4.61, p = .04). These findings demonstrate an apparent overlap in specific white matter architecture underlying the normal variance of divergent thinking, openness, and psychotic-spectrum traits, consistent with the idea of a continuum.

## Introduction

Some research reports positive correlations between various definitions of creativity and a diagnosis of psychopathology [1], [2], [3], [4]. Other studies report that psychopathology is rarely, if ever, associated with creative insight, capacity, or productivity [5]. When artists are studied more carefully, certain personality characteristics appear to reside upon a continuum of both normal behavior and psychopathology. For example, creative expression in the visual arts and poetry has been linked with the overlapping personality traits of schizotypy and Openness to Experience (Openness), and particularly to self-reports of “unusual experiences” and “unconventional nonconformity”, but not the “introvertive anhedonia” characteristic of schizophrenia [6].

Diffusion Tensor Imaging (DTI) offers increased resolution compared to conventional structural MRI regarding white matter microstructure by measurement of water diffusion through cellular compartments *in vivo* [7]. Compared to more isotropic movement of water in gray matter, water diffusion in white matter moves anisotropically, meaning that water diffuses preferentially along the length of the axon compared to perpendicular to the axon. This anisotropic diffusion of water appears to be due to the highly structured axonal membranes and their associated myelin sheaths [8]. By tracking the diffusion of water in the brain a measure of *fractional anisotropy* (FA) can be derived. Higher FA suggests greater axonal coherence and myelination [7], increasing in a roughly linear manner that conforms to normal developmental brain processes [9], [10]. Measures of fractional anisotropy (FA) are considered to be overall measures of axonal integrity, reflecting either increased axonal caliber, increased myelin thickness, increased fiber coherence in a given direction, or some combination of these factors [11].

Both schizophrenic and bipolar patients have been shown to have reduced fractional anisotropy (FA) in the anterior thalamic radiation [12], [13] and uncinate fasciculus [14] within frontal brain regions. Similarly, reduced FA was observed within the uncinate fasciculus of a cohort with schizotypal personality disorder, providing strong support for the hypothesis that similar neural phenotypes may not result in full-blown clinical symptoms [15]. Finally, in normal subjects, the Neuroregulin-1 (NRG1) single nucleotide polymorphisms (SNP's) SNP8NRG243177 and SNP8NRG221533 were found to predict lower FA in the left anterior thalamic radiation [16]. As NRG1 has been found to predict higher risk of schizophrenia [17], [18] and bipolar disorder [19], and is linked with axonal myelination and migration [20], these authors hypothesize a mechanistic link between NRG1 within the anterior thalamic radiation and risk for psychotic disorders [16].

In laboratory settings, creativity is often assessed by tests of divergent thinking (DT), the cognitive process by which one extrapolates many possible answers to an initial stimulus or target data set [21]. We have previously demonstrated that a composite measure of divergent thinking (DT) ability exhibited significant *inverse* relationships with both cortical thickness in frontal lobe regions and metabolite concentration of N-acetyl-aspartate (NAA) in the anterior cingulate cortex in normal young subjects [22], [23]. Here we examine whether white matter integrity, assessed by Fractional Anisotropy (FA), is related to two measures of creativity (Divergent Thinking and Openness to Experience). If a continuum exists between creativity and psychopathology, we hypothesize significant FA-DT relationships within the anterior thalamic radiation and uncinate fasciculus, regions wherein FA reductions have been linked to diagnoses of [12], [13], [14], and higher genetic risk for psychotic disorders [16].

## Methods

### Sample

This study was conducted according to the principles expressed in the Declaration of Helsinki. The study was approved by the Institutional Review Board of the University of New Mexico IRB#05096. All subjects provided written informed consent for the collection of samples and subsequent analysis. Seventy-two volunteers with no history of neurological or psychological disorder participated. Subjects were young adults (22.1+/−2.9 years; 40 males, 32 females); males and females did not differ significantly on Full-Scale Intelligence Quotient (FSIQ), Divergent Thinking (DT), Creative Achievement Questionnaire (CAQ), or Openness. They were recruited by postings in various departments and classrooms around the University of New Mexico. Prior to entry into the study, participants were screened by a clinical neuropsychologist (REJ) and met no criteria for neurological and psychological disorders that would impact experimental hypotheses (e.g., learning disorders, traumatic brain injury, major depressive disorder). Subjects were screened by a computerized Structural Clinical Interview for DSM Disorders (SCID), and followed with a psychological interview to discuss whether any endorsed items rose to the level of clinical diagnoses. Subjects were also screened for conditions that would prohibit undergoing an MRI scan (e.g., metal implant, orthodontic braces, severe claustrophobia).

### Behavioral Measures

1. Four DT tasks were administered: Verbal and Drawing Creativity Tasks, Uses of Objects Test (UOT), described in detail elsewhere [24], [25], and generation of captions to New Yorker Magazine cartoons. In caption generation, subjects are instructed to create as many humorous captions as they can in five minutes for each of three New Yorker Magazine cartoons. They are then instructed to select their “preferred” caption (which was rated) for each at the end of five minutes. All of these tasks tap DT, the aspect of creativity best assessed in laboratory settings.Four independent judges (three females, one male) ranked the DT products of each participant using the consensual assessment technique [26] from which a “composite creativity index” (CCI) was derived. The raters were of the same cohort as the subjects (19–29; college student/graduate). Raters were instructed to rate from 1 (lowest creativity) to 5 (highest creativity) each subjects' DT product according to their own notion of “creativity”, and instructed to bin subjects to conform to a normal distribution (e.g., 5% each 1's and 5's, 10% each 2's and 4's, 70% 3's). Rankings for each subject were averaged across the three measures to form the CCI. The raters had excellent inter-rater reliabilities across the four measures of DT (i.e., CCI α = .81).
2. The Wechsler Abbreviated Scale of Intelligence (WASI) was used to assess intellectual functioning. The WASI consists of four subtests: Vocabulary and Similarities, which produce a Verbal Intelligence Quotient (VIQ), and Block Design, and Matrix Reasoning, which produce a Performance Intelligence Quotient (PIQ). Based on all four subtests one can derive the Full Scale Intelligence Quotient (FSIQ).
3. Personality was assessed with the NEO-FFI, a self-administered measure of normal personality functioning, which produces summary scores across five domains: neuroticism, extraversion, openness, agreeableness, and conscientiousness.

### Diffusion Tensor Imaging Acquisition

Structural imaging was obtained using a 3 Tesla Siemens MRI using a 12-channel head coil. Subjects' heads were stabilized with tape across the forehead and padding around the sides. Echoplanar imaging was acquired: [TE 84 ms; TR 9000; voxel size 2×2×2mm^3^; 72 slices; Field of View = 256mm; 30 diffusion directions with b = 800 s/mm^2^, and 5 measurements with b = 0, acquisition time 5:42]. The DTI experiment was repeated twice, the data was concatenated into one 4D data set, and a concatenated table of corresponding b-value and gradient direction tables was also calculated. The gradient direction vectors corrected for image orientation are stored in the Siemens dicom files and extracted by the dicom2nii program (www.sph.sc.edu/comd/rorden/dicom.html).

All image registrations were done by first extracting the brain using the bet2 (FSL) program. The diffusion tensor, scalar diffusion parameters (Axial Diffusivity – AD, Radial Diffusivity – RD, and FA) were calculated by dtifit (FSL). The FA image was aligned to a FA template with a nonlinear registration algorithm, FNIRT (FMRIB's Nonlinear Image Registration Tool; FSL). This non-linear transformation was applied to AD, and RD images.

### Diffusion Tensor Imaging Analysis

All images were visually inspected for quality, and four subjects were excluded from the analysis due to significant movement or other artifacts (e.g., warping), leaving a final sample of seventy-two. Track Based Statistics (TBSS) allows measurement of water movement along white matter tracts for the entire brain to allow for group analyses of individual subjects on a voxel-by-voxel basis [27]. The fractional anisotropy (FA) image of each subject was normalized to a 1×1×1mm3 FA template in the Montreal Neurological Institute (MNI) space using the non-linear registration algorithm FNIRT/FSL (www.fmrib.ox.ac.uk/fsl/). A mean FA image was calculated from the spatially normalized images of each subject and was “skeletonized” to reflect common tracts across all subjects. FA images of each subject were then projected on the mean FA skeleton. Voxelwise cross-subject statistical analyses were performed using the general linear model in conjunction with 5000 Monte Carlo simulations. To avoid choosing an arbitrary cluster threshold, we used Threshold-Free Cluster Enhancement (TFCE) for final voxelwise inference [28].

### Statistical Analysis

We correlated CCI and Openness scores with FA across all subjects using FSL's General Linear Model (GLM) tool (www.fmrib.ox.ac.uk/fsl/fsl/list). Age, sex, and FSIQ were entered into the model as nuisance variables. All presented results are corrected p-values at p<.05 after controlling for family wise error rate. The correlation inferences were tested using permutation methods with FSL's Randomise (www.fmrib.ox.ac.uk/fsl/randomise). We ran 5000 two-tailed Monte Carlo permutation tests, (i.e., both positive and negative associations) for each of the correlations between CCI, Openness, and FA. Next, we created a mask image for significant FA clusters by binarizing the FA image for results that were significant at p<.05. To determine which diffusion component was driving FA-CCI and FA-Openness relationships, we then used this masked imaged to run the same GLM procedure for the AD, and RD images within voxels that showed significant FA correlations. To avoid choosing an arbitrary initial cluster-forming threshold, we used Threshold-Free Cluster Enhancement (TFCE) for final voxelwise inference. Height (H) was set at 2 and cluster Extent (E) was set at .5 [28].

## Results

Behavioral characteristics of the experimental sample, including measures of creativity (CCI), intelligence (FSIQ), and personality (NEO-FFI), are presented in Table 1. FSIQ and CCI were significantly correlated (r_(IQ,CCI)_ = .34, p = .004), as were Openness and CCI (r_(CCI,Open)_ = .39, p = .001), consistent with previous research [29], and reflecting good psychometric qualities of our “creativity” measure. Intercorrelations between the CCI, FSIQ, and the five components of the NEO-FFI are presented in Table 2.

**Table 1:** 

	Mean	s.d.	Range
CCI	2.9	.43	1.9–3.9
FSIQ	119.9	12.8	86–143
Neuroticism	16.7	8.7	0–43
Extroversion	31.0	6.0	9–45
Openness	34.5	6.5	17–45
Agreeableness	32.4	6.5	12–44
Conscientiousness	33.0	5.8	17–45

CCI = Composite Creativity Index.

FSIQ = Full Scale Intelligence Quotient.

**Table 2:** 

	CCI	FSIQ	N	E	O	A	C
CCI	1						
FSIQ	.34**	1					
Neuroticism	.12	−.08	1				
Extroversion	−.10	−.23	−.20	1			
Openness	.39**	.11	.20	.10	1		
Agreeableness	−.03	.06	−.34**	.22	.05	1	
Conscientiousness	−.06	−.08	−.08	.00	.06	.25*	1

CCI = Composite Creativity Index.

FSIQ = Full Scale Intelligence Quotient.

**Figure 1:**
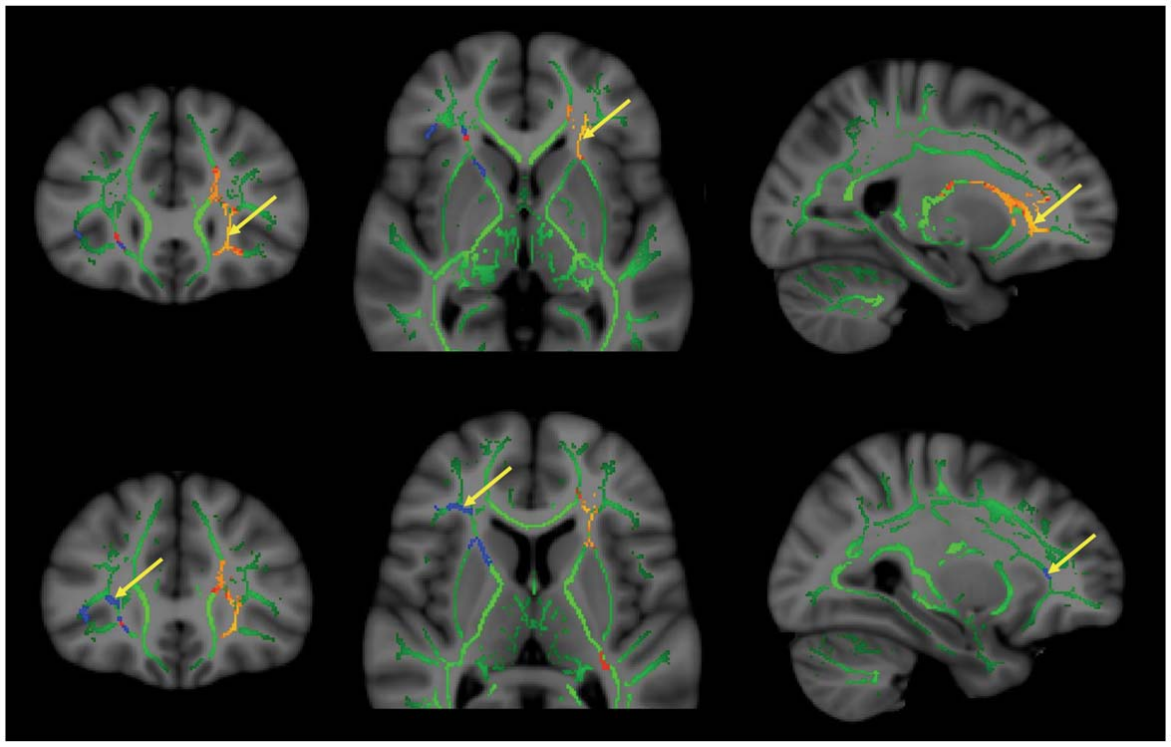
Significant clusters where CCI (Orange/Red; upper panel) and Openness (Blue; lower panel) were inversely related to FA. Arrows indicate maximal significant regions in coronal plane (left), axial plane (middle), and sagittal plane (right). Green regions indicate “skeleton” white matter tracts in which statistical relationships were explored between behavioral variables (CCI/Openness) and white matter integrity (FA). Figures are presented in radiological convention – left side = right hemisphere.

Figure 1 (upper panels) shows regions in which FA was inversely related to CCI (p<.05), with arrows pointing to MNI coordinate (in coronal, axial, and sagittal planes respectively) of the maximally significant cluster within the left anterior thalamic radiation.

Table 3 (upper) shows regions in which CCI was significantly inversely related to FA, RD, and AD within numerous regions within the left hemisphere, especially the anterior thalamic radiation. There was a small region within the right anterior thalamic radiation where the CCI was inversely related to FA. Openness was significantly inversely related to FA within the right anterior thalamic radiation. (p<.05), with arrows pointing to the MNI coordinate (in coronal, axial, and sagittal planes respectively) of the maximally significant cluster within the right inferior frontal white matter/anterior thalamic radiation (Figure 1 lower panels). Table 3 (lower) shows regions in which Openness was significantly inversely related to FA, RD, and AD within a region localized to right anterior thalamic radiation. There were no clusters where positive correlations were found.

**Table 3:** 

**CCI and FA**						
**# of Voxels**	**Corrected Cluster p-value**	**MNI-x**	**MNI-y**	**MNI-z**	**t-value of peak voxel**	**Approximate Tract of peak voxel**
2164	0.011	−23	29	0	5.3639	Left ATR
68	0.045	22	29	−5	4.2447	Right ATR
**CCI and RD**						
**# of Voxels**	**Corrected Cluster p-value**	**MNI-x**	**MNI-y**	**MNI-z**	**t-value of peak voxel**	**Approximate Tract of peak voxel**
2068	0.001	−17	19	−17	2.3012	Left ATR
24	0.046	−28	−29	7	2.3373	Left Retrolenticular IC
3	0.049	−28	−34	11	2.2872	Left Retrolenticular IC
**CCI and AD**						
**# of Voxels**	**Corrected Cluster p-value**	**MNI-x**	**MNI-y**	**MNI-z**	**t-value of peak voxel**	**Approximate Tract of peak voxel**
430	0.014	−23	29	2	2.766	Left ATR
291	0.009	−25	8	20	1.3139	Left ATR
**Openness and FA**						
**# of Voxels**	**Corrected Cluster p-value**	**MNI-x**	**MNI-y**	**MNI-z**	**t-value of peak voxel**	**Approximate Tract of peak voxel**
276	0.04	29	31	8	4.6091	Right ATR
202	0.04	24	13	14	3.7429	Right ATR
61	0.047	20	19	−1	3.9719	Right ATR
**Openness and RD**						
**# of Voxels**	**Corrected Cluster p-value**	**MNI-x**	**MNI-y**	**MNI-z**	**t-value of peak voxel**	**Approximate Tract of peak voxel**
248	0.001	31	33	6	3.878	Right ATR
202	0.001	24	17	12	3.3002	Right ATR
**Openness and AD**						
**# of Voxels**	**Corrected Cluster p-value**	**MNI-x**	**MNI-y**	**MNI-z**	**t-value of peak voxel**	**Approximate Tract of peak voxel**
35	0.014	24	19	10	3.7278	Right ATR
30	0.02	35	35	5	3.738	Right ATR
6	0.039	17	15	−3	3.519	Right ATR

CCI = Composite Creativity Index.

MNI-x = Montreal Neurological Institute – x plane coordinate.

MNI-y = Montreal Neurological Institute – y plane coordinate.

MNI-z = Montreal Neurological Institute – z plane coordinate.

ATR = Anterior Thalamic Radiation.

IC = Internal Capsule.

**Figure 2:**
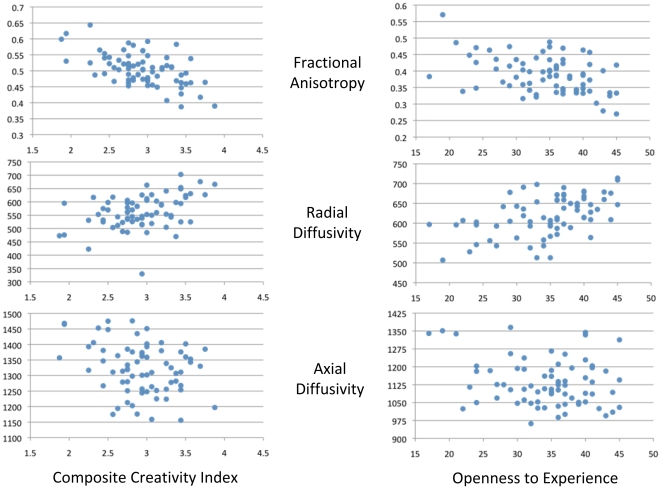
Scatterplots demonstrating relationships between DTI measures and the Composite Creativity Index (left) and Openness to Experience scale from the NEO-FFI (right). Upper panels shows fractional anisotropy (FA) correlations from the maximally significant cluster within the anterior thalamic radiation for CCI (left) and Openness (right); middle panels show radial diffusivity (RD) correlations for the same maximal cluster point; lower panels show axial diffusivity (AD) correlations for the same maximal cluster point.

Figure 2 demonstrates scatterplots within the maximally significant clusters obtained from the anterior thalamic radiation for CCI (left) and Openness (right). The upper panel shows individual subject data for FA; the middle shows RD, and the lower panel shows AD. Post hoc analyses (Table 3) revealed that the major contribution, in terms of significant cluster size, of the FA-CCI and FA-Openness relationships was driven by RD as compared to AD contribution.

## Discussion

Our results suggest a convergence between a cognitive measure of divergent thinking, a personality measure of Openness, and a white matter integrity measure within the inferior frontal lobes. We found that normal young subjects with lower levels of FA within predominantly left inferior frontal white matter (i.e., regions overlapping the uncinate fasciculus and anterior thalamic radiation) scored higher on the CCI; similarly subjects with lower levels of FA within the right frontal white matter (i.e., regions overlapping the uncinate fasciculus and anterior thalamic radiation) scored higher on self-reported measures of Openness. These two regions of white matter overlap with those reported by other researchers who found lower FA in both schizophrenia and bipolar disorder [13], [14], [30]. Subsequent analyses within these regions, designed to elucidate whether axonal or myelin components were driving FA-CCI and FA-Openness relationships tended to show relatively greater RD contribution within frontal white matter regions. Previous studies have indicated RD decrements linked to myelin as opposed to axonal diffusivity [31], suggesting lower levels of myelination in subjects scoring higher on our measures of Openness and CCI.

The anterior thalamic radiations include association fibers linking the thalamus to projection zones within the prefrontal cortices, in one of many functionally segregated thalamo-fronto-striatal loops [32]. Interestingly, researchers have found that lower FA was “unrelated to age, duration of illness, current measures of psychopathology, prescribed medication, or the presence of psychotic symptoms” in bipolar and schizophrenia disorders, with implications that “white matter connectivity represents a disease or vulnerability related feature common to both disorders” [14]. In contrast to the current frontal and predominantly left lateralized CCI-FA findings, our previous morphometry study of creativity found generally posterior and right lateralized CCI-thickness relationships [22]. Researchers studying morphometric correlates of personality found an inverse correlation between thickness and Openness limited to the right inferior parietal cortex (i.e., angular gyrus) in an elderly cohort [33]. With so few studies in the extant literature utilizing morphometric measures and measures of Openness or DT, it is difficult to link systematically our DTI findings with these previous findings other than to note the general inverse brain-behavior nature of the relationships across research groups. Future studies in large normal cohorts undertaken with both DTI and cortical thickness measures, would further elucidate potential brain networks involved in creative cognition and personality. Similarly, measures of creative cognition and personality (e.g., DT and Openness) should be administered in tandem with DTI measures, both to patients diagnosed with psychotic disorders (e.g., schizophrenia, bipolar) and their unaffected relatives, to better understand the causal mechanisms underlying lower FA, creativity, and psychosis within frontal white matter.

The neural processes involving creative cognition can be included in the realm of optimal brain functioning. The present findings suggest a complex picture of “optimal” white matter microstructure in health—especially when the white matter correlates of DT and intelligence appear in direct opposition [34], [35]. Similarly disparate results were evident in our previous studies showing that DT ability was associated with both increased *and* decreased cortical thickness at various loci [22], and results from MR spectroscopy showing that N-acetyl-aspartate and DT relationships may be direct or inverse—depending on whether IQ falls above or below 120 [23]. These findings of “less is sometimes more,” broadly consistent with the notion of “neural efficiency,” were evident in the earliest functional studies of intelligence [36], and have subsequently been supported in both intelligence [37] and creativity neuroimaging studies [38]. A picture begins to emerge regarding the manifestation of complex cognitive processes in the brain, including the interplay of intelligence and creativity. Whereas more neural resources are often associated with higher intellectual capacity in a parieto-frontal network of brain regions [39], studies in DT appear to suggest that less is often better in a different network of brain regions, particularly fronto-cingulate-subcortical networks linked via white matter loops [40].

There are several important limitations for interpreting results. First, there does not exist one “creativity”; rather, this construct is hypothesized to reside upon a continuum between cognitive (i.e., scientific) and emotional (i.e., artistic) behavioral domains [41], [42]. Thus, when comparing scientists and artists directly, researchers have found lower lifetime rates of psychopathology for: 1) scientists compared to artists, 2) natural scientists compared to social scientists, 3) nonfiction writers compared to fiction writers and poets, and 4) formal artists compared to “expressive” artists [3], [4], [43]. These findings have led researchers to hypothesize a hierarchical structure of creativity across disciplines [42], which echoes the notions of “paradigmatic” (i.e., a fundamental model of events) versus “revolutionary” (i.e., rejection of doctrines) approaches as applied to the sciences [44]. The benefits of working within the lines of a given field appear to be lower levels of psychopathology; alternately, individuals with lower levels of psychopathology may be attracted to such endeavors. Similarly, there is increasing evidence that the cost of “revolutionary” approaches to creative endeavors, whether it is in the arts or sciences, may be associated with increased levels of psychopathology although, again, causative links are weak at best.

Second, measures of DT are only proxy measure of one aspect of creativity, as we and numerous other researchers have noted [5], [22], [29], with this measure “having to do with fluency of thinking and flexibility of thinking, abilities concerned with the ready flow of ideas and with readiness to change direction or to modify information” [21]. If the definition of creativity, as widely accepted, involves a tension between novelty and usefulness, then measures of DT appear to pull for aspects of novel cognitive production amenable to laboratory settings. Viewed within the context of the hierarchical construct of creative endeavors reviewed above, this tension plays out among creative actors by means of their chosen profession (more or less disciplined) and their role within that profession (more or less conforming) [42]. Thus, DT measures would generally appear to bias measurement of novelty over usefulness, would value measurement of less disciplined and conforming responses (as scored by our judges), and would be expected to have a higher overlap with psychopathology than creativity measures biasing “usefulness” (e.g., Creative Achievement Questionnaire) based on the incidence of psychopathology across creative professions [43].

Finally, DTI measures themselves lack the level of specificity desired to determine subtle overlap between patient and normal cohorts. Indeed, measures of FA represent a conglomeration of ratio measures reflecting diffusion of water along the axon and perpendicular to the axon, the combination of which is considered to be a proxy measure for axonal health. However, while lower FA is commonly seen in diseases where both cognition and white matter integrity are impaired (e.g., Traumatic Brain Injury, Schizophrenia, Alzheimer's disease) [45], [46], [47], evidence is accumulating that higher FA in particular brain regions may also be associated with clinical disorders including post-traumatic stress disorder [48], obsessive-compulsive disorder [49], panic disorder [50], synaesthesia [51], and Williams syndrome [52]. We have attempted to provide additional specificity to our findings by including measures of RD and AD, but the general point remains that optimal white matter integrity, as quantified by measures of FA, RD, and AD may underpin both cognitive functioning and clinical disease status. Recent cross-sectional evidence showing a curvilinear relationship between age and measures of FA, AD, and RD would tend to support the notion of optimal regional white matter integrity [53], [54].

The current research suggests that lower FA within the inferior frontal white matter is associated with increased DT (left lateralized) and increased Openness (right lateralized), highlighting that lower FA within these brain regions is not specific to psychopathology nor to creative expression on DT tasks. Further research is required; however, the current results support the notion that clinical psychosis may be an extreme manifestation of neurobehavioral fitness traits exhibiting normal variation within human social cognition [6], [55], [56]. All of these factors remind us that the brain is complex: there is little likelihood of finding one-to-one correspondence between regional FA findings and cognitive, personality, or clinical constructs. The apparent overlap in FA findings between creativity and psychopathology is consistent with there being a continuum of creativity and psychopathology but the overlap may also reflect 1) limitations in how researchers measure creativity (i.e., over-reliance on DT measures), 2) conflation of creative expression across individuals (i.e., paradigmatic versus revolutionary), 3) and recent appreciation of the continuous (versus categorical) nature of clinical symptoms in the manifestation of psychopathology [57].
